# Personality and oral health-related quality of life. Results from an online survey

**DOI:** 10.1186/s12903-022-02486-7

**Published:** 2022-11-02

**Authors:** André Hajek, Hans-Helmut König

**Affiliations:** Department of Health Economics and Health Services Research, University Medical Center Hamburg-Eppendorf, Hamburg Center for Health Economics, Hamburg, Germany

**Keywords:** Oral health, Oral health-related quality of life, Personality, Neuroticism, Conscientiousness

## Abstract

**Background:**

To investigate the association between personality factors and oral health-related quality of life.

**Methods:**

Data were taken from an online survey (representative for the general adult population in Germany in terms of region, sex and age group; n = 3,075) performed in late summer 2021. The well-established Oral Health Impact Profile (OHIP-G5) was used to measure oral health-related quality of life. Moreover, the established 10 Item Big Five Inventory (BFI-10) was used to quantify personality factors (in terms of agreeableness, conscientiousness, extraversion, neuroticism, and openness to experience). Sex, age, family status, educational level, smoking status, alcohol consumption, sports activities, presence of chronic diseases and self-rated health were adjusted for in multiple linear regression analysis.

**Results:**

Pearson correlations between oral health-related quality of life and personality factors ranged from r =- 0.17 (conscientiousness) to r = 0.17 (neuroticism). Regressions revealed that low oral health-related quality of life is associated with higher neuroticism (β = 0.39, p < 0.001) and lower conscientiousness (β=-0.51, p < 0.001).

**Conclusion:**

This study revealed an association between personality factors (higher neuroticism and lower conscientiousness) and low oral health-related quality of life. Before dental treatment, it may be helpful to measure personality traits of patients in order to predict the expectations of patients, as well as their responses to intended treatments. This may support the identification of the most appropriate method of treatment.

**Supplementary Information:**

The online version contains supplementary material available at 10.1186/s12903-022-02486-7.

## Background

Oral health-related quality of life refers to perceived oral symptoms, as well as the experienced functional and psychosocial impacts of oral disorders [1]. It is an important component of the overall health of individuals. A lower oral health-related quality of life can contribute to feelings of loneliness, social isolation or lower mental health [2, 3]. It is also associated with morbidity and mortality [4].

For example, prior research has shown that sociodemographic factors such as age and educational level are associated with oral health-related quality of life.

To date, various studies have examined the determinants of oral health-related quality of life [5, 6]. For example, previous research revealed that low oral health-related quality of life was associated with lower income and lower education [5, 6], as well as with age [7]. Moreover, an association between lifestyle factors and oral health-related quality of life has been shown [8]. Similarly, an association between health-related factors and oral health-related quality of life has been found [9, 10]. However, to date, there is limited knowledge regarding the association between personality factors and oral health-related quality of life [11–18]. Personality factors commonly refer to the “big five”: agreeableness (referring to altruism, and compliant behavior), conscientiousness (tendency to be goal-directed and to be careful), extraversion (tendency to be outgoing), neuroticism (referring to feelings of anxiety or depression, being impulsive) and openness to experience (tendency to be open to new ideas and experiences). For example, previous studies showed an association between higher neuroticism and lower oral health-related quality of life ([12, 15–18]), and also showed an association between higher conscientiousness and higher oral health-related quality of life [11]. However, most existing studies are limited in terms of their small sample sizes, illness-specific samples, or specific age groups (e.g., individuals in old age). Therefore, our goal was to assess the correlates of oral health-related quality of life in a general population sample. Individuals at risk of poor oral health-related quality of life may benefit from such knowledge. Moreover, assessing personality factors is important as it can predict patient behavior during treatment [17]. Thus, examining personality factors in association with oral health-related quality of life is potentially important for the therapeutic strategy of clinicians [19, 20]. Furthermore, personality traits may play a role in determining individual limits of oral health-related quality of life for specific treatments [17]. Personality factors can contribute to patients’ satisfaction with therapy [20]. In sum, the aim of this current study was to clarify the association between personality factors and oral health-related quality of life among the general adult population in Germany.

### Sample

Data for our study were gathered from a nationally representative online survey of individuals aged 18 to 70. All of the participants were from Germany. Younger individuals (aged 17 years or lower) and older individuals (aged 71 years and above), as well as individuals not residing in Germany, were excluded. Around 14,000 individuals received an invitation to participate (response rate approximately 22%). The total sample size was n = 3,075. There were no missing values for the variables examined in this study. For reasons of data availability, we could not compare non-participants and participants (for example, with regard to age bracket or sex).

The survey took place from late August to early September 2021. The participants were recruited by a well-known market research firm (respondi). An online sample was used in such a way that it matched the gender, age, and federal state distributions in the German population [21]. A comparison between our sample and the target cohort in terms of gender, state and age group is given in Supplementary Table 1.

### Outcome

To measure oral health-related quality of life, the Oral Health Impact Profile (OHIP-G5) was used [22]. It addresses the following four areas: Oral function, orofacial pain, appearance, and psychosocial impact. It has good to very good psychometric characteristics [22]. For example, individuals were asked (in each case: 0-never, 1-hardly ever, 2-occasionally, 3-fairly often, and 4-very often): Have you felt that there has been less flavor in your food because of problems with your teeth, mouth, dentures or jaws?

This tool runs from 0 to 20, with higher scores corresponding to a *lower* oral health-related quality of life. Cronbach’s alpha was 0.85 in our study.

### Independent variables

Our key independent variables were related to personality characteristics. To measure personality (in terms of agreeableness, conscientiousness, extraversion, neuroticism and openness to experience), the 10-item Big Five Inventory (BFI-10) [23] was used. It is a well-established personality inventory that assesses the five major personality dimensions (with two items each; each dimension ranges from 1 to 5; a higher score reflects a more pronounced personality factor).

In regression analysis, we adjusted for some potential confounders (in accordance with prior research [2] and also based on theoretical considerations): sex, age, marital status (four categories: married, living together with spouse; married, not living together with spouse; divorced; widowed; single), educational level (upper secondary school; qualification for applied upper secondary school; polytechnic Secondary School; intermediate Secondary School; Lower Secondary School; currently in school training/education; without school-leaving qualification), labor force participation (full-time employed; retired; other), smoking behavior (never smoker; no, not anymore; yes, sometimes; yes, daily), alcohol consumption (daily; several times per week; once a week; 1–3 times per month; less often; never) and sports activities (regularly, more than 4 h a week; regularly, 2–4 h a week; regularly, 1–2 h a week; less than one hour a week; no sports activity), self-rated health (single item from 1 = very bad to 5 = very good) and chronic disease (no chronic disease; one or more chronic diseases). For example, prior research has shown that sociodemographic factors such as age and educational level are associated with oral health-related quality of life [7]. Moreover, an association between lifestyle factors and oral health-related quality of life has been shown [8]. Similarly, an association between health-related factors and oral health-related quality of life has been found [9, 10].

### Statistical analysis

Sample characteristics were calculated. Subsequently, Pearson correlations between personality factors and oral health-related quality of life were calculated. Lastly, multiple linear regressions were conducted to investigate the association between personality factors and oral health-related quality of life.

Mean variance inflation factor (VIF) was 1.36 (highest VIF was 1.99) indicating that multicollinearity was not a threat. Based on the Breusch-Pagan test we examined whether heteroscedasticity was present. Based on the results (Chi²= 427.17, p < 0.001), we can reject the null hypothesis of constant variance – implying the presence of heteroscedasticity in the residuals. For this reason, we calculated cluster-robust standard errors. Moreover, we used standardized normal probability plots to check the normality of residuals. The residuals have an approximately normal distribution following such plots.

The level of significance was set at p < 0.05. For performing statistical analyses, Stata 16.1 (Stata Corp., College Station, Texas) was used.

## Results

### Sample characteristics and bivariate analysis

Sample characteristics are shown in Table 1. Average age was 44.5 years (from 18 to 70 years; SD: 14.8 years) and 48.9% were male. Mean OHIP-G5 score equaled 2.2 (ranging from 0 to 20; SD: 3.3). Average agreeableness score was 3.1 (1 to 5; SD: 0.8), average conscientiousness score was 3.7 (1 to 5; SD: 0.8), average extraversion score was 3.1 (1 to 5; SD: 1.0), average neuroticism score was 2.7 (1 to 5; SD: 1.0) and average openness to experience score was 3.3 (1 to 5; SD: 1.0).

**Table 1:** Sample characteristics

**Variables**	**Mean (SD) / N (%)**
Oral health-related quality of life (OHIP-G5) (from 0 to 20, with higher scores indicating lower oral health-related quality of life)	2.2 (3.3)
Sex	
Men	1502 (48.8%)
Women	1570 (51.1%)
Diverse	3 (0.1%)
Age	44.5 (14.8)
Marital status	
Single / Divorced / Widowed / Married, not living together with spouse	1313 (42.7%)
Married, living together with spouse	1762 (57.3%)
Highest educational degree	
upper secondary school	1326 (43.1%)
qualification for applied upper secondary school	328 (10.7%)
polytechnic Secondary School	168 (5.5%)
intermediate Secondary School	888 (28.9%)
lower Secondary School	347 (11.3%)
currently in school training/education	9 (0.3%)
without school-leaving qualification	9 (0.3%)
Employment status	
Full-time employed	1458 (47.4%)
Retired	499 (16.2%)
Other	1118 (36.4%)
Smoking	
Yes, daily	716 (23.3%)
Yes, sometimes	251 (8.2%)
No, not anymore	843 (27.4%)
Never smoker	1265 (41.1%)
Sports activities	
No sports activity	834 (27.1%)
Less than one hour a week	629 (20.5%)
Regularly, 1–2 h a week	714 (23.2%)
Regularly, 2–4 h a week	473 (15.4%)
Regularly, more than 4 h a week	425 (13.8%)
Alcohol intake	
Daily	186 (6.0%)
Several times per week	564 (18.3%)
Once a week	495 (16.1%)
1–3 times per month	532 (17.3%)
Less often	715 (23.3%)
Never	583 (19.0%)
Chronic diseases	
Absence of chronic diseases	1765 (57.4%)
Presence of at least one chronic disease	1310 (42.6%)
Self-rated health (1 = very bad to 5 = very good)	3.6 (0.9)
Agreeableness (from 1 to 5, with higher values indicating higher levels of agreeableness)	3.1 (0.8)
Conscientiousness (from 1 to 5, with higher values indicating higher levels of conscientiousness)	3.7 (0.8)
Extraversion (from 1 to 5, with higher values indicating higher levels of extraversion)	3.1 (1.0)
Neuroticism (from 1 to 5, with higher values indicating higher levels of neuroticism)	2.7 (1.0)
Openness to experience (from 1 to 5, with higher values indicating higher levels of openness to experience)	3.3 (1.0)

Pearson correlations between personality factors (1. agreeableness, 2. conscientiousness, 3. extraversion, 4. neuroticism, and 5. openness to experience) and oral health-related quality of life were as follows: (1) r=-0.05, p < 0.01; (2) r=-0.17, p < 0.001; (3) r=-0.05, p < 0.01; (4) r = 0.17, p < 0.001; (5) r=-0.01, p = 0.66.

### Regression analysis

Results of multiple linear regressions are given in Table 2. R² was 0.11.

**Table 2:** Association between personality factors and oral health-related quality of life. Results of multiple linear regressions

Independent variables	Oral health-related quality of life
Agreeableness	-0.06
	(0.07)
Conscientiousness	-0.51***
	(0.08)
Extraversion	0.04
	(0.06)
Neuroticism	0.39***
	(0.07)
Openness to experience	0.03
	(0.06)
Potential confounders	✓
Observations	3,075
R²	0.11

Unstandardized beta-coefficients are displayed; cluster-robust standard errors in parentheses; *** p < 0.001, ** p < 0.01, * p < 0.05, + p < 0.10; Potential confounders include sex, age, family status, educational level, presence of currently smoking, alcohol consumption, sports activities, presence of chronic diseases and self-rated health

Adjusting for various covariates, linear regressions revealed that low oral health-related quality of life is associated with higher neuroticism (β = 0.39, p < 0.001) and lower conscientiousness (β=-0.51, p < 0.001). In contrast, low oral health-related quality of life was not associated with agreeableness (β=-0.06, p = 0.43), extraversion (β = 0.03, p = 0.55) and openness to experience (β = 0.03, p = 0.56).

## Discussion

Drawing on data from a large, nationally representative sample, our objective was to investigate the association between personality factors and oral health-related quality of life. Regressions revealed that low oral health-related quality of life is associated with higher neuroticism and lower conscientiousness, but is not associated with the other three personality factors (i.e., agreeableness, extraversion and openness to experience). Using data from the general adult population, our current study greatly extends our current knowledge on the link between personality factors and oral health-related quality of life.

The results from regression analysis support previous findings regarding an association between higher neuroticism and lower oral health-related quality of life [12, 15–18]. Furthermore, the results from regression analysis confirm previous research regarding an association between higher conscientiousness and higher oral health-related quality of life [11]. More broadly, such findings are in accordance with previous studies showing an association between lower neuroticism/higher conscientiousness and favorable health outcomes such as lower frailty scores [24], better functional abilities [25], fewer depressive symptoms [26], better self-rated health [27] as well as favorable lifestyle factors such as not smoking, lower alcohol intake and non-obesity [28–30].

The association between higher conscientiousness and higher oral health-related quality of life may be explained by the fact that individuals scoring high in conscientiousness frequently use health-promotion activities [31] and have regular dental visits [32]. Consequently, they may report a higher oral health-related quality of life. Moreover, such a behavior (e.g., in terms of diligence, orderliness or self-discipline) is associated with both presence and high count of natural teeth [33]. A previous systematic review showed that tooth loss is associated with lower oral health-related quality of life [34]. It should be repeated that low conscientiousness is associated with poor lifestyle habits such as smoking [29] – which is also predictive of tooth loss [35].

A possible explanation for the association between higher neuroticism and lower oral health-related quality of life may be that individuals scoring high in neuroticism may affirm negative symptoms and may feel pain more intensely [18]. Such individuals are commonly very aware of their oral health and may very critically evaluate their oral health-related quality of life [18]. Furthermore, they often report dental anxiety [36]. Additionally, they are also often unsatisfied with dental treatment [20] compared to individuals scoring low in neuroticism. As mentioned above, it has been shown that higher neuroticism is associated with lower self-rated health (which is associated with oral health-related quality of life [37]) both cross-sectionally and longitudinally [27]. Prior research has also shown that lower optimism (which is associated with higher neuroticism [38]) is associated with lower oral health-related quality of life [39] which may assist in explaining the findings of this study. Moreover, higher neuroticism is associated with lower well-being [40] and higher negative affect [41]. Lower well-being [42] and higher negative affect [43] in turn are associated with lower oral health-related quality of life.

It should be noted that Pearson correlations between some personality characteristics (agreeableness, extraversion and openness) and oral health-related quality of life were small in terms of effect size. Moreover, the Pearson correlation between openness and oral health-related quality did not achieve statistical significance. While the sign for two associations ([1] between extraversion and oral health-related quality of life; [2] between openness and oral-health related quality of life) changed from the Pearson correlation to multiple linear regression, it should be emphasized that both associations did not achieve statistical significance in regression analysis.

Some strengths and limitations are worth acknowledging. We examined the association between personality and oral health-related quality of life based on data from a representative online survey. An established instrument was used to measure oral health-related quality of life. Moreover, the BFI-10 was used to quantify personality traits. Nevertheless, more sophisticated tools (regarding oral health-related quality of life and personality factors) are required to investigate this association in further detail. Moreover, this is a cross-sectional study with its known limitations (e.g., regarding causality). Furthermore, data were taken from an online sample. Thus, some selection bias (in terms of online affinity) may be present. Additionally, the response rate was quite low. Moreover, our study focused on individuals aged 18 to 70 years. Thus, future research regarding the association between personality factors and oral health-related quality of life among children/adolescents and oldest old individuals is needed.

## Conclusions and future research

This study revealed an association between personality factors (higher neuroticism and lower conscientiousness) and low oral health-related quality of life. Assessing personality factors prior to dental treatment may be useful in predicting patients’ expectations of, and their responses to, available treatments. This can assist in determining the best treatment options. With regard to future research, longitudinal studies in particular are required to confirm the current findings.

## Electronic supplementary material

Below is the link to the electronic supplementary material.

Supplementary Table

## Data Availability

The datasets generated and/or analysed during the current study are not publicly available due to ethical restrictions but are available from the corresponding author on reasonable request.

## References

[1] Locker D, Allen F (2007). What do measures of ‘oral health-related quality of life’ measure?. Community Dent Oral Epidemiol.

[2] Hajek A, König H-H (2022). Oral health-related quality of life, probable depression and probable anxiety: evidence from a representative survey in Germany. BMC Oral Health.

[3] Hajek A, König H-H (2021). The Association between Oral Health-Related Quality of Life, Loneliness, Perceived and Objective Social Isolation—Results of a Nationally Representative Survey. Int J Environ Res Public Health.

[4] Heitmann BL, Gamborg M (2008). Remaining teeth, cardiovascular morbidity and death among adult Danes. Prev Med.

[5] Makhija SK, Gilbert GH, Boykin MJ, Litaker MS, Allman RM, Baker PS (2006). The Relationship Between Sociodemographic Factors and Oral Health–Related Quality of Life in Dentate and Edentulous Community-Dwelling Older Adults. J Am Geriatr Soc.

[6] Soldera EB, Ortigara GB, Bonzanini LI, Schulz RE, Danesi CC, Antoniazzi RP (2020). Clinical and sociodemographic factors associated with oral health-related quality of life in survivors of head and neck cancer. Head Neck.

[7] Rebelo MAB, Cardoso EM, Robinson PG, Vettore MV (2016). Demographics, social position, dental status and oral health-related quality of life in community-dwelling older adults. Qual Life Res.

[8] Silva AE, Demarco FF, Feldens CA (2015). Oral health–related quality of life and associated factors in S outhern B razilian elderly. Gerodontology.

[9] Jensen PM, Saunders RL, Thierer T, Friedman B (2008). Factors associated with oral health–related quality of life in community-dwelling elderly persons with disabilities. J Am Geriatr Soc.

[10] Baniasadi K, Armoon B, Higgs P, Bayat AH, Mohammadi Gharehghani MA, Hemmat M (2021). The Association of Oral Health Status and socio-economic determinants with Oral Health‐Related Quality of Life among the elderly: A systematic review and meta‐analysis. Int J Dent Hyg.

[11] Gabriella D, Klemens R, Xiao-Hui RF, Corinna B, Eva H (2021). Effect of personality traits on the oral health-related quality of life in patients with oral lichen planus undergoing treatment. Clin Oral Investig.

[12] Lin F, Ye Y, Ye S, Wang L, Du W, Yao L (2018). Effect of personality on oral health-related quality of life in undergraduates. Angle Orthod.

[13] Ishida K, Nogawa T, Takayama Y, Saito M, Yokoyama A (2017). Does Neuroticism Influence Oral Health-Related QOL in Patients with Removable Partial Dentures?. JDR Clin Trans Res.

[14] Montero J, Gómez-Polo C (2017). Association Between Personality Traits and Oral Health-Related Quality of Life: A Cross-Sectional Study. Int J Prosthodont.

[15] Soeda H, Sato Y, Yamaga E, Minakuchi S (2017). A structural equation model to assess the influence of neuroticism on oral health-related quality of life in complete denture wearers. Gerodontology.

[16] Takeshita H, Ikebe K, Kagawa R, Okada T, Gondo Y, Nakagawa T (2015). Association of personality traits with oral health-related quality of life independently of objective oral health status: a study of community-dwelling elderly Japanese. J Dent.

[17] Fädler A, Hartmann T, Bernhart T, Monshi B, Rappersberger K, Hof M (2015). Effect of personality traits on the oral health-related quality of life in patients with oral mucosal disease. Clin Oral Investig.

[18] Al-Omiri MK, Karasneh J (2010). Relationship between oral health-related quality of life, satisfaction, and personality in patients with prosthetic rehabilitations. J Prosthodont.

[19] Boye B, Lundin KE, Leganger S, Mokleby K, Jantschek G, Jantschek I (2008). The INSPIRE study: do personality traits predict general quality of life (Short form-36) in distressed patients with ulcerative colitis and Crohn’s disease?. Scand J Gastroenterol.

[20] Ra’ed Omar AH, Mahmoud Khalid AO, Ahed Mahmoud AW (2006). Psychological impact on implant patients’ oral health-related quality of life. Clin Oral Implants Res.

[21] Münnich R, Gabler S. 2012: Stichprobenoptimierung und Schätzung in Zensus 2011. Wiesbaden: Statistisches Bundesamt; 2012.

[22] John MT, Miglioretti DL, LeResche L, Koepsell TD, Hujoel P, Micheelis W (2006). German short forms of the oral health impact profile. Community Dent Oral Epidemiol.

[23] Rammstedt B, John OP (2007). Measuring personality in one minute or less: A 10-item short version of the Big Five Inventory in English and German. J Res Pers.

[24] Hajek A, Kretzler B, König H-H (2021). Relationship between personality factors and frailty. A systematic review. Arch Gerontol Geriatr.

[25] Hajek A, König HH (2021). Personality and functional impairment. Evidence from the Survey of Health, Ageing and Retirement in Europe. Psychogeriatrics.

[26] Hakulinen C, Elovainio M, Pulkki-Råback L, Virtanen M, Kivimäki M, Jokela M (2015). Personality and depressive symptoms: Individual participant meta‐analysis of 10 cohort studies. Depress Anxiety.

[27] Stephan Y, Sutin AR, Luchetti M, Hognon L, Canada B, Terracciano A (2020). Personality and self-rated health across eight cohort studies. Soc Sci Med.

[28] Hakulinen C, Elovainio M, Batty GD, Virtanen M, Kivimäki M, Jokela M (2015). Personality and alcohol consumption: Pooled analysis of 72,949 adults from eight cohort studies. Drug Alcohol Depend.

[29] Hakulinen C, Hintsanen M, Munafò MR, Virtanen M, Kivimäki M, Batty GD (2015). Personality and smoking: Individual-participant meta‐analysis of nine cohort studies. Addiction.

[30] Jokela M, Hintsanen M, Hakulinen C, Batty GD, Nabi H, Singh-Manoux A (2013). Association of personality with the development and persistence of obesity: a meta‐analysis based on individual–participant data. Obes Rev.

[31] Bogg T, Roberts BW (2004). Conscientiousness and health-related behaviors: a meta-analysis of the leading behavioral contributors to mortality. Psychol Bull.

[32] Aarabi G, Walther C, Bunte K, Spinler K, Buczak-Stec E, König H-H, et al. The Big Five personality traits and regularity of lifetime dental visit attendance: evidence of the Survey of Health, Ageing, and Retirement in Europe (SHARE). Aging Clin Exp Res. 2021. 10.1007/s40520-021-02051-2 PMC9151578 34964080

[33] Mõttus R, Starr JM, Deary IJ (2013). Predicting tooth loss in older age: Interplay between personality and socioeconomic status. Health Psychol.

[34] Gerritsen AE, Allen PF, Witter DJ, Bronkhorst EM, Creugers NH (2010). Tooth loss and oral health-related quality of life: a systematic review and meta-analysis. Health Qual Life Outcomes.

[35] Bakri N, Tsakos G, Masood M (2018). Smoking status and oral health-related quality of life among adults in the United Kingdom. Br Dent J.

[36] Vassend O, Røysamb E, Nielsen CS (2011). Dental anxiety in relation to neuroticism and pain sensitivity. A twin study. J Anxiety Disord.

[37] Benyamini Y, Leventhal H, Leventhal EA (2004). Self-rated oral health as an independent predictor of self-rated general health, self-esteem and life satisfaction. Soc Sci Med.

[38] Sharpe JP, Martin NR, Roth KA (2011). Optimism and the Big Five factors of personality: Beyond neuroticism and extraversion. Pers Individ Dif.

[39] Brennan DS, Spencer AJ (2012). Social Support and Optimism in Relation to the Oral Health of Young Adults. Int J Behav Med.

[40] Soto CJ (2015). Is happiness good for your personality? Concurrent and prospective relations of the big five with subjective well-being. J Pers.

[41] Kroencke L, Geukes K, Utesch T, Kuper N, Back M (2020). Neuroticism and emotional risk during the COVID-19 pandemic. J Res Pers.

[42] Locker D, Ma C, Payne B (2000). Self-perceived oral health status, psychological well-being, and life satisfaction in an older adult population. J Dent Res.

[43] Brennan DS, Singh KA, Spencer AJ, Roberts-Thomson KF (2006). Positive and negative affect and oral health-related quality of life. Health Qual Life Outcomes.

